# Metabolomics Reveal Induction of ROS Production and Glycosylation Events in Wheat Upon Exposure to the Green Leaf Volatile Z-3-Hexenyl Acetate

**DOI:** 10.3389/fpls.2020.596271

**Published:** 2020-12-03

**Authors:** Maarten Ameye, Lieven Van Meulebroek, Bianca Meuninck, Lynn Vanhaecke, Guy Smagghe, Geert Haesaert, Kris Audenaert

**Affiliations:** ^1^Laboratory of Applied Mycology and Phenomics, Department of Plants and Crops, Faculty of Bioscience Engineering, Ghent University, Ghent, Belgium; ^2^Laboratory of Agrozoology, Department of Plants and Crops, Faculty of Bioscience Engineering, Ghent University, Ghent, Belgium; ^3^Laboratory of Chemical Analysis, Department of Veterinary Public Health and Food Safety, Faculty of Veterinary Medicine, Merelbeke, Belgium

**Keywords:** green leaf volatile, metabolomic, wheat, fusarium, oxidative stress

## Abstract

The activation and priming of plant defense upon perception of green leaf volatiles (GLVs) have often been reported. However, information as to which metabolic pathways in plants are affected by GLVs remains elusive. We report the production of reactive oxygen species in the tip of young wheat leaves followed by activation of antioxidant-related enzyme activity. In this study, we aimed to uncover metabolic signatures upon exposure to the GLV Z-3-hexenyl acetate (Z-3-HAC). By using an untargeted metabolomics approach, we observed changes in the phenylpropanoid pathways which yield metabolites that are involved in many anti-oxidative processes. Furthermore, exposure to GLV, followed by infection with *Fusarium graminearum* (Fg), induced significantly greater changes in the phenylpropanoid pathway compared to a sole Z-3-HAC treatment. Fragmentation of a selection of metabolites, which are significantly more upregulated in the Z-3-HAC + Fg treatment, showed D-glucose to be present as a substructure. This suggests that Z-3-HAC induces early glycosylation processes in plants. Additionally, we identified the presence of hexenyl diglycosides, which indicates that aerial Z-3-HAC is metabolized in the leaves by glycosyltransferases. Together these data indicate that GLV Z-3-HAC is taken up by leaves and incites oxidative stress. This subsequently results in the modulation of the phenylpropanoid pathway and an induction of glycosylation processes.

## Introduction

The production of biogenic volatile organic compounds is one of the defense strategies that plants mount at the advent of an attack by a pathogen or insect or by wounding (Dudareva et al., 2013). Green leaf volatiles (GLVs) are quickly (within seconds to minutes) produced upon biotic stress and can subsequently be perceived by neighboring plants (Fall et al., 1999; Ameye et al., 2018). GLVs have often been described to play an important role in the defense of plants against deleterious insects (Turlings et al., 1991; Scala et al., 2013), pathogens (Nakamura and Hatanaka, 2002; Kishimoto et al., 2008) and abiotic stress (Yamauchi et al., 2015) by direct activation or by priming for enhanced defense (Engelberth et al., 2004; Ameye et al., 2015; Dombrowski et al., 2019). Being produced upon mechanical or biotic damage, GLVs have been assigned a role as long distance damage-associated molecular patterns, eliciting defense response (Quintana-Rodriguez et al., 2018). Recently, another defensive role for GLVs has been uncovered: GLV esters induce stomatal closure in several crops, thereby preventing infection by pathogens, as was shown for *Pseudomonas syringae* pv. *tomato* DC3000 in tomato (López-Gresa et al., 2018).

Despite its widespread occurrence throughout the plant kingdom, it remains enigmatic via which mechanism GLV perception and signal transduction occur (Heil, 2014; Ameye et al., 2018). However, there is evidence that GLVs influence the redox status of plants and hence induce plant stress responses (Cofer et al., 2018). Among the early reactions are plasma membrane potential depolarization, followed by an increase in cytosolic [Ca^2+^] which, in turn, activates respiratory burst oxidase homologs (Asai et al., 2009; Zebelo et al., 2012). A transcriptomics study showed that Z-3-HOL induced the expression of genes involved in transcriptional regulation, lipid signaling, and cell wall reinforcement in maize (Engelberth et al., 2013). In *Arabidopsis*, it has been reported that, following exposure to GLV E-2-hexenal, one-third of the upregulated genes were unique and unrelated to plant hormones (Mirabella et al., 2015).

In a previous work, we have demonstrated that pre-exposure of wheat to Z-3-hexenyl acetate (Z-3-HAC) leads to enhanced defense against the hemi-biotrophic fungus *Fusarium graminearum* (Schwabe), a causal agent of the cereal disease fusarium head blight (Ameye et al., 2015). This enhanced defense coincided with a suppression of salicylic acid (SA)-related defense during the biotrophic phase of the fungus at 24 h after inoculation (hai) and an increase in JA-related defense during the necrotrophic phase of the fungus at 48 hai. However, to better understand which defense mechanisms were influenced upon Z-3-HAC exposure, a more detailed analysis of earlier plant responses is necessary.

Omics techniques, such as proteomics and metabolomics, are indispensable to bridge the genotype–phenotype gap (Patti et al., 2012; Feussner and Polle, 2015; Hong et al., 2016). Whereas proteins can be modified post-translationally, metabolites represent intermediary and downstream biochemical products and serve as signatures of metabolic pathways (Patti et al., 2012). The application of metabolomics in plant physiology is gaining traction. However, because of the lack of annotated databases, metabolite identification remains a major limitation for non-targeted plant metabolomics (Creek et al., 2014; Feussner and Polle, 2015; Razzaq et al., 2019). Consequently, current phytometabolome pathways are mostly restricted to genome-reconstructed pathways (Kind et al., 2009; Feussner and Polle, 2015). Due to this bottleneck, many studies have, in addition to untargeted analysis, also focused on how plant metabolites from *a priori* chosen pathways change in response to pathogen infections (Urano et al., 2009; Ward et al., 2010; Mhlongo et al., 2016).

As plant–pathogen interactions consist of a tightly regulated dynamic and transient activation of defense pathways, an analysis of the early effects of Z-3-HAC on plant defense is designated to uncover the changes in the plant metabolome. Using an untargeted metabolomics approach, we aimed to reveal hitherto unknown priming mechanisms by Z-3-HAC, which may aid in better understanding how the plants’ metabolome changes after perceiving GLVs from the environment and how biological functionalities are controlled.

## Materials and Methods

### Z-3-HAC Exposure Experiment

To test the effect of Z-3-HAC on plant health and reactive oxygen species (ROS) production, wheat seeds (var. Sahara, AVEVE, Belgium) were sterilized in NaOCl (14%) for 5 min and rinsed three times with distilled water and placed in 1.5 L glass jars filled with 250 mL growth medium [4.4 g L^–1^ MS salts with vitamins (Duchefa Biochemie, Haarlem, Netherlands), 10 g L^–1^ sucrose, and 8 g L^–1^ plant agar (Duchefa Biochemie, Haarlem, Netherlands)] at pH 5.7. After 1 week, the plants were exposed to 50 μM (corresponding to 5.9 g m^–3^) Z-3-HAC by pipetting 9.9 μL on a piece of sterile filter paper placed inside the pot, followed by closing the lid and sealing it off with PTFE tape. At 0, 1, 2, 6, and 8 h after exposure (hae), the plants were removed from the jars, and multispectral images were taken.

### Multispectral Imaging and Histochemical Staining

To automatically measure the effect of Z-3-HAC exposure on the health of wheat seedlings, we employed a custom-built phenotyping platform equipped with a multispectral camera (CropReporter, Phenovation, Wageningen, Netherlands). The color images and the maximum efficiency of PSII (*F*_v_/*F*_m_) (Baker, 2008) were measured according to the manufacturer’s specifications. To detect H_2_O_2_ accumulation, staining by 3,3-diaminobenzidine (DAB) was executed according to the protocol of Thordal-Christensen et al. (1997), with minor modifications from De Vleesschauwer et al. (2010).

### RT-qPCR and Enzyme Assay

The expression of genes involved in ROS production and quenching was measured using RT-qPCR. RNA from the leaf sheaths was extracted using TRI reagent (Sigma-Aldrich) according to the manufacturer’s specifications and quantified with a fluorometer (Quantus, Promega Benelux, Leiden, Netherlands). First-strand complementary DNA was synthesized from 500 ng of total RNA using the Goscript kit (Promega Benelux, Leiden, Netherlands). The primers used for quantitative reverse transcription (qRT)-PCR analysis are listed in Supplementary Table 1. RT-qPCR analysis was performed using a CFX96 system (Bio-Rad). The thermal profile consisted of an initial denaturation step for 2 min at 95°C, followed by 40 cycles of 95°C for 15 s and 60°C for 60 s. Finally, melting curve analysis was performed using a temperature profile of 95°C for 10 s, cooling to 65°C for 5 s, and subsequently heating to 95°C at a rate of 0.5°C per 10 s. Normalization of wheat defense genes was carried out using cell division control protein (Ta54227) and actin (Ta35284) as reference genes (Paolacci et al., 2009). All calculations and analyses of the quality of the reference genes were performed using qBase + software (Biogazelle, Zwijnaarde, Belgium).

Superoxide dismutase (706002, Cayman Chemical) and catalase enzyme activity (70002, Cayman Chemical) and phenolic content (K527, BioVision) were measured using colometric assays according to the manufacturer’s specifications. Enzyme activity and phenolic content were normalized to the amount of protein measured according to the manufacturer’s specifications (Roti Nanoquant, Carl Roth GmbH, Karlsruhe, Germany).

### Sample Preparation for UHPLC–HRMS/MS

A total of 100 mg of 6–8 pooled leaf sheaths from the leaf sheath assay was crushed using liquid nitrogen. Afterward, 1 mL of cold (−20°C) modified Bieleski extraction buffer consisting of methanol, ultrapure water, and formic acid (75:20:5, v/v/v) was added. Additionally, the suspension was amended with a deuterium-labeled internal standard of 100 pg μL^–1^ d_6_-abscisic acid (OlChemIm, Olomouc, Czech Republic). The samples were vortexed and placed at −20°C for 12 h of cold extraction. The samples were centrifuged for 10 min at 10,000 rpm, and 500 μL of the supernatant was transferred to a 30 kDa Amicon^®^ Ultra centrifugal filter unit (Merck, Millipore Corporation, MA, United States). Finally, the extract was transferred to a high-performance liquid chromatography (HPLC) vial. Samples of different treatments were placed in the ultra-HPLC coupled to high-resolution tandem mass spectrometry (UHPLC–HRMS/MS) system in a randomized manner. Finally, 10 μL was injected directly on the column for UHPLC–HRMS/MS analysis. Two quality control (QC) samples were prepared by using aliquots of 10 randomly chosen samples, representing the different treatments. QC samples were run after each set of 10 samples and were used to correct for possible chromatographic and mass spectrometric variations.

### UHPLC–HRMS/MS Analysis

The UHPLC–HRMS/MS system consisted of an Dionex UltiMate 3000 XRS UHPLC pumping system (Thermo Fisher Scientific, San Jose, CA, United States), coupled to a Q-Exactive^TM^ hybrid quadrupole-Orbitrap mass spectrometer (Thermo Fisher Scientific, San Jose, CA, United States), being equipped with a heated electrospray ionization source (HESI-II) that was operated in switching polarity mode. The instrumental parameters for HESI-II ionization and mass spectrometric detection can be found in Van Meulebroek et al. (2012). Chromatographic separation of the compounds was achieved with a gradient elution program using a reversed phase Nucleodur Gravity C18 column (1.8 μm, 50 mm × 2.1 mm ID) (Macherey-Nagel, Düren, Germany). The mobile phase consisted of a binary solvent system: 0.1% formic acid in ultrapure water (solvent A) and methanol (solvent B) at a constant flow rate of 300 μL min^–1^. A linear gradient profile with the following proportions (v/v) of solvent A was applied: 0–1 min at 98%, 1–2.50 min from 98 to 60%, 2.50–4 min from 60 to 50%, 4–5 min from 50 to 20%, 5–7 min at 20%, 7–7.10 min from 20 to 0%, 7.10–8 min at 0%, and 8–8.01 min from 0 to 98%, followed by 2 min of re-equilibration. The column oven temperature was set at 30°C. Phytohormones were identified based on both the retention time relative to the internal standard and accurate mass (*m/z*) using an analytical standard (Supplementary Table 2) (OlChemIm, Olomouc, Czech Republic). Instrument control and data processing were carried out by Xcalibur 3.1 software (Thermo Fisher Scientific, San Jose, CA, United States). For HRMS-MS, parallel reaction monitoring was used. An isolation window of 0.5 *m/z* was used, and ions were fragmented at 35 eV in the collision cell.

#### Pathway Analysis

In order to get an initial grasp of the pathways which are affected by the different treatments, we employed the mummichog algorithm (Li et al., 2013). This algorithm permitted to functionally characterize metabolites and map metabolite features from our HPLC-MS analysis to current metabolic models and to assess the significance and enrichment of metabolic pathways. For this, we used the *MS Peaks to Pathways* module in MetaboAnalyst (V4.0) (Chong et al., 2018). The molecular weight tolerance was set at 5 ppm. The *P*-value cutoff was 0.01, and the Kyoto Encyclopedia of Genes and Genomes (KEGG) pathway library was chosen, with *Oryza sativa japonica* as the model organism for monocotylydonous plants.

#### Chemometric Data Analysis

To identify metabolites which are differentially produced upon Z-3-HAC exposure, we employed the chemometrics strategy previously described in Van Meulebroek et al. (2015). A first step concerned the characterization of detected metabolite features in terms of retention time, *m/z* value, and abundance along the various samples. To this end, Sieve^TM^ 2.1 (Thermo Fisher Scientific, San Jose, CA, United States) was used. Full-scan data were provided as input, and the following settings were applied: an *m/z* range of 100–800 Da, an *m/z* width of 5 ppm, a retention time range of 1.5–9.0 min, a peak intensity threshold of 10^6^ arbitrary units, a maximum peak width of 0.5 min, and a maximum number of 15,000 frames. This step was preceded by a peak alignment process to correct for inherent chromatographic variability, thereby allowing a maximum retention time shift of 0.2 min. This step rendered 4,310 metabolite features for the positive ionization mode and 1,145 for the negative ionization mode.

Metabolite features and their abundances were used to construct predictive models, implementing multivariate data analysis techniques such as principal component analysis (PCA) and orthogonal partial least squares discriminant analysis (OPLS-DA) using SIMCA^TM^ 15 software (Umetrics, Malmö, Sweden). Hereby Pareto scaling and log transformation were applied to standardize the range of independent X variables and induce normality, respectively. For OPLS-DA, supervised models were established to classify samples according to their treatment and time points (Table 1). Model validity was tested with CV-ANOVA (*P* < 0.05) and permutation testing (*n* = 100). Additionally, according to Triba et al. (2015), a good OPLS-DA model should have Q^2^(Y) > 0.5. It was concluded that the OPLS-DA models, as obtained for the positively ionized fingerprints, were valid and showed good predictability, which was not the case for the models that were constructed based on the negatively ionized metabolite features. In the following step, we selected the metabolites that contributed most to the predictability of the model by evaluating the variable importance in projection (VIP) scores (> 1), Jack-knifed confidence intervals (not across zero), and S-plots (| correlation *p*(corr)| > 0.5 and | covariance *p*| > 0.04) (Supplementary Figure 5). As such, we initially retained 103 metabolite features. We further narrowed down this selection to a final number of 13 unique metabolites representing the highest VIP and S-plot scores. Hereby we also assessed whether a particular peak was not wrongly assigned various feature identity labels by SIEVE^TM^ and whether isotopes or different adducts of the same metabolite were present. Metabolites that were only important at a single time point were also omitted from further analysis.

**TABLE 1:** Orthogonal partial least squares discriminant analysis (OPLS-DA) models based on the metabolite features from the positive ionization mode provide better predictability compared to the negative ionization mode.

**Time**	**Ionization mode**	**Model characteristics**					
		**Number of principal components (predictive + orthogonal)**	***R*^2^ (X)**	***R*^2^ (Y)**	***Q*^2^ (Y)**	**CV-ANOVA (*P*-value)**
1 hai	+	1 + 2	0.624	0.99	0.976	4.95 × 10^–6^
	-	1 + 2	0.786	0.913	0.57	0.222
6 hai	+	1 + 2	0.633	0.998	0.972	2.82 × 10^–5^
	-	1 + 2	0.572	0.837	0.576	0.128
24 hai	+	1 + 2	0.721	0.996	0.936	0.0169
	-	1 + 2	0.742	0.992	0.837	0.098

**Model characteristics for the different OPLS-DA models of Z-3-HAC + F. graminearum-treated plants vs. F. graminearum-treated plants at 1, 6, and 24 h after inoculation (hai).**

#### Identification

Tentative identification of the retained selection of metabolites was performed using different data sources, i.e., isotope pattern, accurate mass (*m/z*), and HRMS/MS fragmentation pattern. For this purpose, Sirius software (Version 4.0.1, Jena, Germany) (Böcker and Dührkop, 2016) and the MetFrag web application (Ruttkies et al., 2016), linked to the public databases ChemSpider (Royal Society of Chemistry), PubChem (National Center for Biotechnology Information), and KEGG (Kyoto Encyclopedia of Genes and Genomes), were used.

### Data Analysis

Data were analyzed and visualized by usage of SPSS (Version 22.0, IBM Corp., Armonk, NY, United States) and R (R Core Team, 2013).

### Infection Assay

In order to study changes in the metabolome upon Z-3-HAC exposure and a subsequent *F. graminearum* infection, unless stated otherwise, four different treatments were used: (1) a control treatment, (2) a treatment in which wheat plants were exposed to Z-3-HAC, (3) a treatment in which Z-3-HAC-exposed wheat plants were subsequently challenged with a conidia suspension of *F. graminearum*, and (4) a treatment in which non-exposed plants were challenged with a conidia suspension of *F. graminearum*.

*F. graminearum* 8/1, containing a green fluorescent protein coding gene (Jansen et al., 2005), was used in this study. The strain was grown on potato dextrose agar for 10 days at 21°C under a regime of 12 h of dark and 12 h of combined UVC and UVA light (2 × TUV 8W T5 and 1 × TL 8W BLB; Philips). Macroconidia were harvested by adding 0.01% (v/v) Tween 80 to the Petri dishes and rubbing the mycelium with a sterile Drigalski spatula. Afterward, the suspension was adjusted to a final concentration of 10^5^ conidia mL^–1^.

Two weeks old winter wheat seedlings (var. Sahara) were exposed overnight to Z-3-HAC using a custom-built exposure system previously described in Ameye et al. (2015). The open flow system consisted of four nalophan bags which were supplied with purified air. In two of the bags (treatment 2 + 3), 70 μL of pure Z-3-HAC (Sigma-Aldrich) was applied to a piece of filter paper, thus avoiding direct contact with the plant. In the two other bags (treatment 1 + 4), distilled water was used. As nalophan bags were consistently flushed, the aerial concentrations of Z-3-HAC reached maximum values of 100 μM, which rapidly declined within minutes. Even though wheat seedlings were exposed to a high concentration of Z-3-HAC, the aerial Z-3-HAC concentration declined very rapidly to previously reported concentrations (Wenda-Piesik et al., 2010; Piesik et al., 2011). As stated by Matsui et al. (2012), aqueous concentrations of GLVs within cells can reach values up to 1 mM. Thus, using a high concentration for a short period of time mimics GLV concentrations in damaged cells (Heil, 2014). On the following morning, the seedlings were taken out of the bags and used in the infection described in Koga et al. (2004). Leaf sheaths were carefully peeled of the stem and subsequently inoculated with 10 μL of a conidia suspension (10^5^ conidia mL^–1^). At different time points, samples were taken and flash-frozen in liquid nitrogen. The samples were stored at −80°C until analysis.

## Results

### ROS Production and Quenching Upon Z-3-HAC Exposure

**FIGURE 1:**
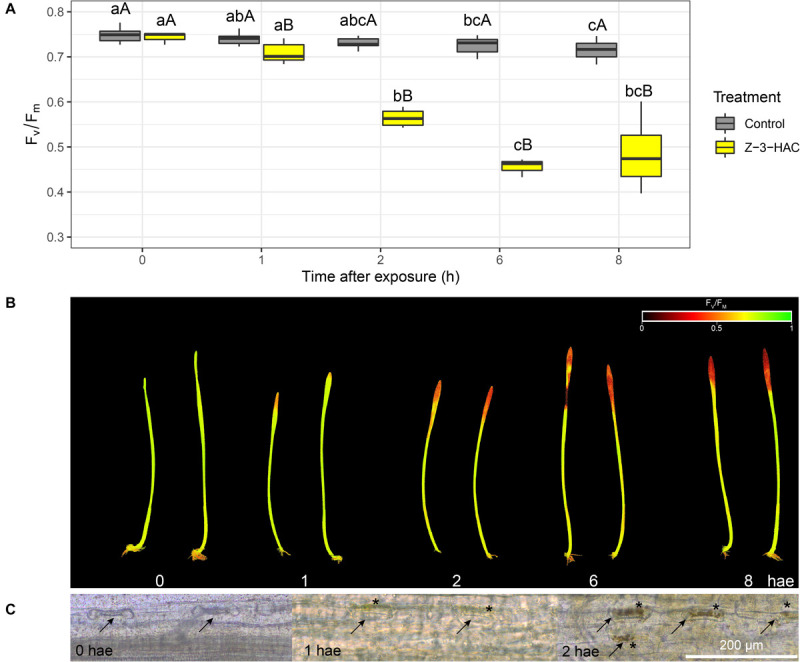
Exposure to 50 μM Z-3-hexenyl acetate results in an initial decline in maximum efficiency of photosystem II (*F*_v_/*F*_m_) in 2 weeks old wheat plants concurrent with an accumulation of H_2_O_2_ around the stomata in the top of the leaves. **(A)** *F*_v_/*F*_m_ values (*n* = 4 per time point) at different time points. Different lowercase letters above the box plots indicate significant differences between the time points per treatment level (Tukey’s HSD test, α = 0.05). Different uppercase letters indicate significant differences between the treatments per time point (two-sample *t*-test, α = 0.05). **(B)** Corresponding images of the Z-3-HAC-treated leaves with the *F*_v_/*F*_m_ values depicted using a continuous color scale. **(C)** Microscopic images of the stomata in which the H_2_O_2_ is shown to accumulate using 3,3-diaminobenzidine (DAB) staining. Arrows indicate the stomata, and asterisks depict locations of DAB accumulation.

**FIGURE 2:**
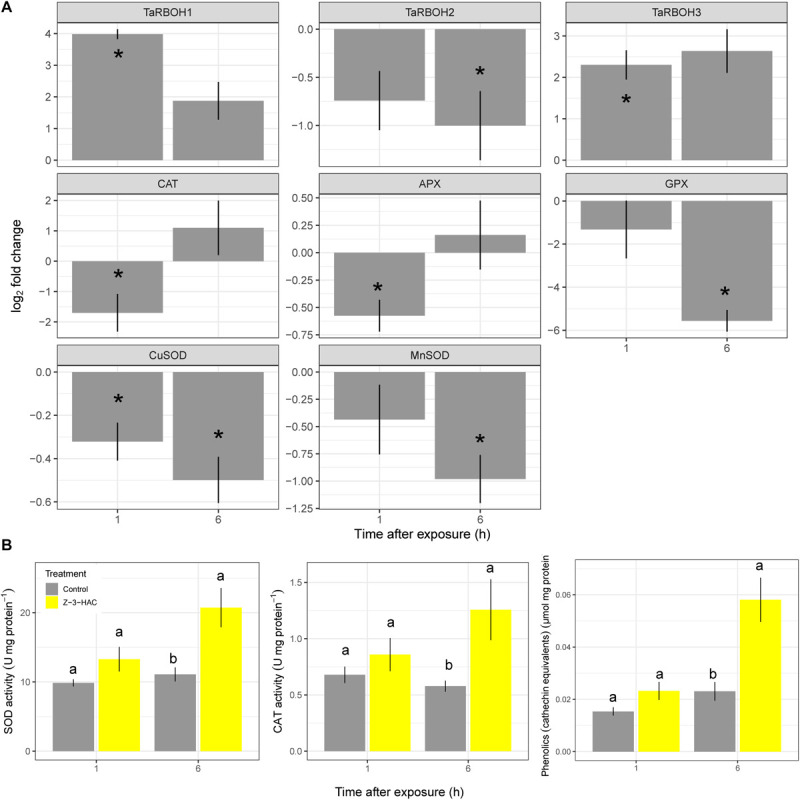
Gene expression **(A)** and enzyme activity **(B)** of processes involved in the production and quenching of reactive oxygen species after exposure to Z-3-HAC. **(A)** Bars represent the log_2_-transformed means of four biological replicates, each consisting of three pooled upper part of leaves showing reductions in *F*_v_/*F*_m_. Fold change was calculated by dividing the CNRQ values of the Z-3-HAC-treated leaves to the control leaves. Asterisks depict significant changes (one-sample *t*-test, α = 0.05). **(B)** Bars represent the means of four biological replicates from the same samples used for the RT-qPCR. Enzyme activity and phenolics content were normalized to the amount of protein present. Different uppercase letters above the box plots indicate significant differences between the time points per treatment (two-sample *t*-test, α = 0.05) Different lowercase letters above the box plots indicate significant differences between the treatments per time point (two-sample *t*-test, α = 0.05).

Wheat seedlings were exposed in a closed environment to Z-3-HAC in order to investigate its effects on *F*_v_/*F*_m_ values as a proxy for plant health. Two-way ANOVA revealed that there was an effect of the treatment (*P* < 0.001) and time after exposure (*P* < 0.001) and that there was an interaction between these factors (*P* < 0.001). Multispectral images revealed that, at 1 hae, *F*_v_/*F*_m_ already started to decline, which continued until 8 hae (Figure 1A), of which the effect was significantly greater in Z-3-HAC-treated plants compared to the control plants. This effect on *F*_v_/*F*_m_ values was only situated at the top of the leaves and does not further migrate downwards in the leaves (Figure 1B). Further microscopic analysis using DAB staining revealed that this decline in *F*_v_/*F*_m_ values could be attributed to H_2_O_2_ accumulation surrounding the stomata which induced oxidative damage (Figure 1C). To examine this further in detail, we performed RT-qPCR (Figure 2A) and enzyme activity assays (Figure 2B) of genes and enzymes involved in ROS production and quenching by the wheat seedlings at 1 and 6 hae. The upper part of the leaf which showed reductions in *F*_v_/*F*_m_ was selected. Genes *TaRBOH1* and *TaRBOH3*, responsible for O_2_^–^ production, had a 16.1-fold (*P* = 0.003) and 5.4-fold (*P* = 0.049) increase at 1 hae and 4.7-fold (*P* = 0.136) and 7.5-fold (*P* = 0.069) at 6 hae, respectively, upon Z-3-HAC exposure. Concurrently, superoxide dismutase *CuSOD* (*P* = 0.010) and *MnSOD* (*P* = 0.011), which react with 2O_2_^–^ to form H_2_O_2_, were significantly downregulated at 6 hae. *CAT*, coding for catalase, which reacts with H_2_O_2_ to form H_2_O and O_2_, was significantly downregulated at 1 hae (*P* = 0.024) (Figure 2A). Additionally, the gene ascorbate peroxidase (*APX*) was downregulated at 1 hae (*P* = 0.003). Glutathione peroxidase (*GPX*) was significantly downregulated at 6 hae (*P* = 0.048) (Figure 2A). When we look at enzyme activity, for SOD, we saw a significant effect of treatment (*P* = 0.006) and time (*P* = 0.044), but no interaction using a two-way ANOVA. *Post hoc* analysis showed that, at 6 hae, SOD activity was significantly higher in the Z-3-HAC treatment compared to the control (+ 86.8%, *P* = 0.036) at 6 hae. The two-way ANOVA for CAT activity revealed a significant effect of the treatment (*P* = 0.02), but not of the time after exposure (*P* = 0.370); also no interaction between the treatment and hae was present (*P* = 0.144). *Post hoc* analysis showed a significantly higher CAT activity (+ 117%, *P* = 0.048) upon Z-3-HAC exposure (Figure 2B) at 6 hae. Additionally, two-way ANOVA of the phenolic content, which is also implicated in the quenching of oxidative stress, revealed an effect of treatment (*P* = 0.001) and time (*P* = 0.001), and an interaction was present between treatment and time (*P* = 0.018). At 6 hae, phenolics were significantly higher in seedlings exposed to Z-3-HAC at 6 hae (+ 152%, *P* = 0.009) compared to the control treatment, and in the Z-3-HAC treatment, phenolic content was higher at 6 hae compared to that at 1 hae (+ 153%, *P* = 0.009) (Figure 2B).

### Untargeted Metabolomics

#### Functional Pathway Analysis Upon Z-3-HAC Exposure

**FIGURE 3:**
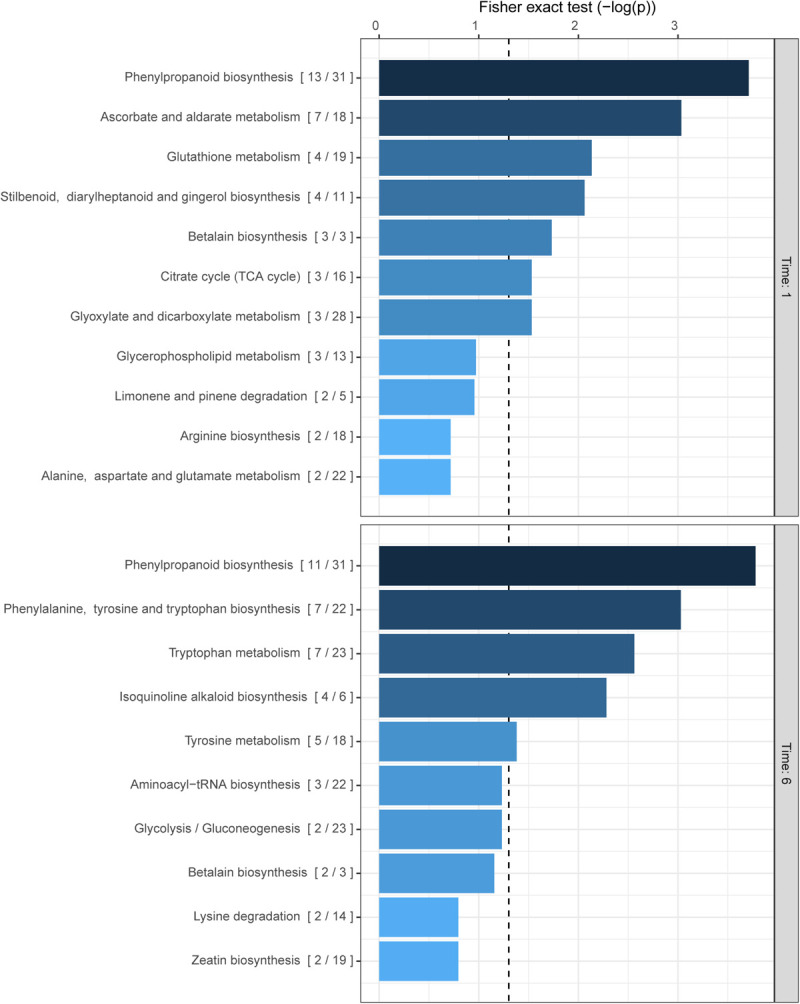
Biochemical pathways in wheat which are affected by exposure to Z-3-HAC after 1 and 6 h. The mummichog algorithm was used on the *m/z* dataset of six biological replicates per treatment per time point. The ratio in brackets represents the number of significant matches to the total number of matched compounds in the pathway. Bars next to pathways represent the *p*-value of the Fisher exact test which represents the significance of change in the pathway following exposure to Z-3-HAC. The dashed line represents a *p*-value of 0.05.

The previous results indirectly show the production of phenolic compounds in response to ROS accumulation. Several groups of plant metabolites have already been reported to be involved in the quenching of oxidative stress in plants such as phenolic compounds, carotenoids (Havaux, 2014), ascorbate (Caverzan et al., 2012), and glutathione (Szalai et al., 2009) among others (Škerget et al., 2005; Dai and Mumper, 2010; Akinwumi et al., 2018). However, a more holistic approach is warranted to elucidate the effects of Z-3-HAC exposure to wheat. Therefore, to uncover more detailed specific changes in the metabolome of wheat seedlings upon exposure to Z-3-HAC, we performed an untargeted metabolomics analysis. For this, the mummichog algorithm was chosen to functionally characterize unknown metabolites and assess which metabolic pathways in wheat were significantly changed. For this experiment, we used an open-flow system previously used in Ameye et al. (2015) to mimic sudden bursts in GLV production following foliar damage. At 1 hae, we observed the highest significant change in the metabolite content of compounds of the phenylpropanoid pathway, followed by ascorbate and aldarate metabolism, glutathione, stilbenoid, diarylheptanoid, and gingerol biosynthesis, and betalain biosynthesis, which are all involved in the quenching of oxidative stress in plants (Figure 3). At 6 hae, the pathways which were significantly up- or down-regulated are involved in the biosynthesis of phenylpropanoids, phenylalanine tyrosine and tryptophan, and isoquinoline alkaloids (Figure 3).

#### Functional Pathway Analysis Upon Z-3-HAC Exposure and *Fusarium graminearum* Infection

**FIGURE 4:**
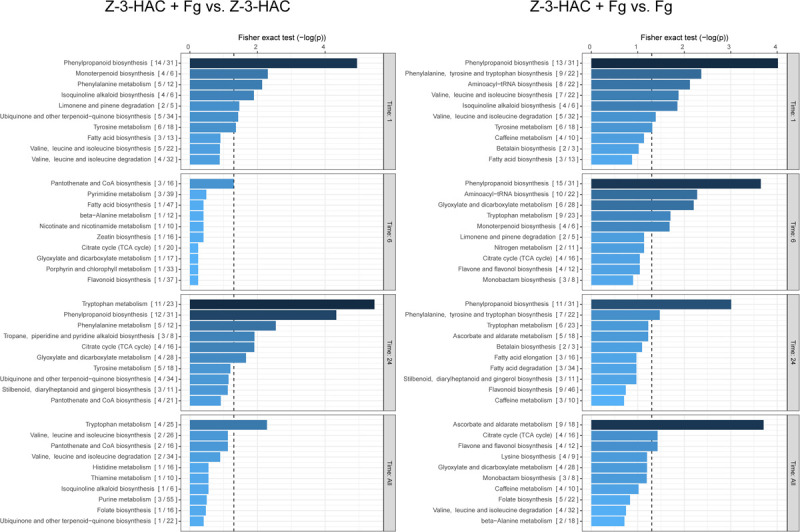
Biochemical pathways in wheat which are affected by exposure to Z-3-HAC after 1, 6, and 24 h after infection. The mummichog algorithm was used on the *m/z* dataset of six biological replicates per treatment per time point. To analyze the added effect of pre-exposure to Z-3-HAC after infection, the *m/z* dataset of the Z-3-HAC + Fg treatment was compared to the *m/z* dataset of the Z-3-HAC treatment on the one hand (left panel) and to the *m/z* dataset of the Fg dataset on the other hand (right panel). Bars next to pathways represent the *p*-value of the Fisher exact test which represents the significance of change in the pathway when two treatments are compared. The ratio in brackets represents the number of significant matches to the total number of matched compounds in the pathway. The dashed line represents a *p*-value of 0.05.

As we were primarily interested in the additional effect of pre-treating plants with Z-3-HAC on the metabolome following an infection with Fg, we compared the dataset of the Z-3-HAC + Fg treatment to the dataset of the Z-3-HAC treatment alone and the Fg treatment alone (Figure 4). This allowed us to distinguish the effects of the sole treatments Z-3-HAC and Fg, respectively, and to identify pathways which were more or less upregulated in plants that were pre-treated with Z-3-HAC and subsequently infected.

At 1, 6, and 24 hai, we observed that metabolites in the phenylalanine pathways and the more downstream phenylpropanoid and tryptophane pathways were significantly affected by the Z-3-HAC + Fg treatment, which goes beyond the separate effects of Z-3-HAC or Fg (Figure 4). To corroborate the observed results with respect to the phenylalanine pathway, we performed a targeted analysis using an analytical standard of L-phenylalanine (L-Phe), the starting product of the pathway. In the Z-3-HAC and Z-3-HAC + Fg-treated seedlings, L-Phe was significantly lower compared to the control and *Fg*-inoculated seedlings at 1 and 6 hai (Supplementary Figure 1). This either suggests a decreased L-Phe production in these treatments or an increased demand of L-Phe from the downstream pathways.

#### Selection and Tentative Identification of Biologically Relevant Metabolites

**FIGURE 5:**
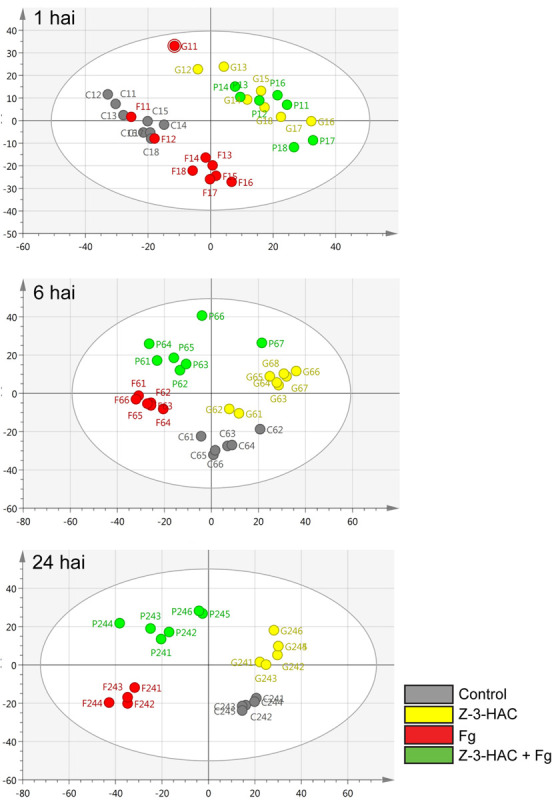
Principal component analysis (PCA) score plots for the different time points. The PCA plots were constructed with metabolome data from the positive ionization mode for different time points: 1, 6, and 24 h after inoculation (hai). Each circle represents a single sample of 6–8 leaf sheaths. Control, gray; Z-3-HAC, yellow; Fg, red; Z-3-HAC + Fg, green. The ellipse depicts the Hotelling’s *T*^2^ 95% confidence interval.

**FIGURE 6:**
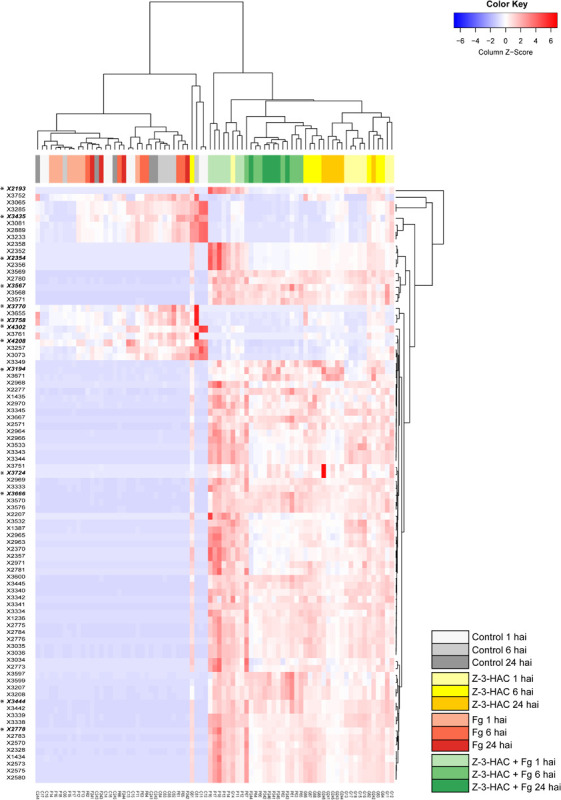
Heat map and complete-linkage hierarchical clustering of the peak areas of the 103 metabolite features (horizontal lines) which contributed most to the orthogonal partial least squares discriminant analysis model for the positive ionization mode. A final set of 13 metabolite features was retained for further analysis, each one marked with an asterisk and in bold. Each vertical line has been assigned a color which corresponds to the treatment and time point of the sample. Metabolite feature codes (identity labels) were automatically assigned by Sieve^TM^ 2.1 software (Thermo Fisher Scientific, San Jose, CA, United States). Z-3-HAC, Z-3-hexenyl acetate; Fg, *Fusarium graminearum*; hai, hours after inoculation.

The mummichog algorithm provided a powerful tool to get a first grasp on the changes in the metabolome related to Z-3-HAC exposure. However, as this strategy is limited to the available metabolites included in the KEGG database, we pursued a more tentative identification approach. First, we performed PCA, whereby the PCA-X score plots were based on 4,310 positively ionized metabolite features and showed that samples from the same treatment cluster together and separate from the other treatments, which became clear from 6 hai onward (Figure 5). This indicated that metabolic differences were present between the various treatments. The total variance explained by the first two principal model components was 45, 56, and 65% for 1, 6, and 24 hai, respectively. OPLS-DA models were constructed in order to reveal those metabolites that contributed to the differentiation between the Z-3-HAC and Z-3-HAC + Fg treatment. Initially, 103 metabolites from the positive ionization mode were retained for further chemical and biological assessment. Complete-linkage hierarchical clustering of the metabolites according to their abundance profiles along samples revealed two large clusters: one cluster in which a metabolite was most abundant in Z-3-HAC and Z-3-HAC + *Fg* treatments and another cluster in which the metabolites were least abundant in the Z-3-HAC + *Fg* treatment (Figure 6). The latter metabolites may be inhibited by Fg infection in Z-3-HAC-treated plants or serve as a substrate for the production of metabolites further downstream.

**FIGURE 7:**
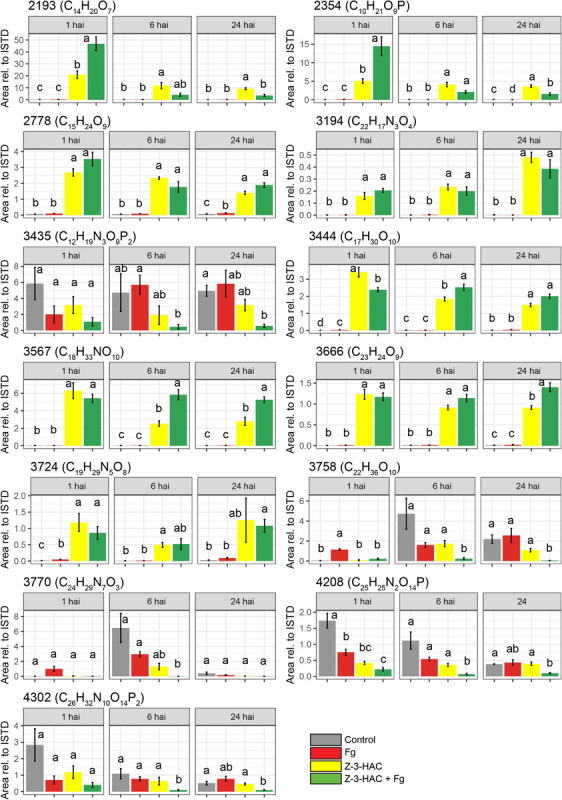
Metabolite features which contribute most to the predictability of the orthogonal partial least squares discriminant analysis model for the positive ionization mode. Bars represent average integrated peak area values relative to the integrated peak area of the internal standard, and this is for 6–8 biological replicates. Treatments: control (gray bars); Fg, *F. graminearum* inoculation (red bars); Z-3-HAC, Z-3-hexenyl acetate exposure (yellow bars); Z-3-HAC + *Fg* inoculation (green bars); hai, hours after inoculation. Error bars represent ± SE. Significant differences between treatments per time point are depicted with different letters. The significance of differences was calculated using *post hoc* Dunett’s T3 test (α = 0.05). Metabolite codes were automatically assigned by Sieve^TM^ 2.1 software (Thermo Fisher Scientific, San Jose, CA, United States).

Metabolites were selected for HRMS/MS fragmentation, and tentative identification was established using full-scan data and the MS/MS patterns. Remarkably, the metabolites that were most abundantly present in the Z-3-HAC-treated plants were tentatively identified as glycosylated compounds (Table 2, Figure 7, and Supplementary Figure 2). Using an analytical standard, we determined the MS/MS pattern of D-glucose (Merck, Darmstadt, Germany) and were able to confirm that various fragments of the metabolites under investigation were matching those of D-glucose, indicating the presence of a glucose group and pointing toward glycosylated metabolites (Supplementary Figure 2 and Supplementary Dataset 1). The metabolites from the other cluster did not possess fragments matching those of D-glucose. Besides the fragments that were shared with those of D-glucose, 30 unique fragment ions were also shared between at least two different metabolites (Supplementary Figure 3), pointing to metabolites that share similar (sub)structures.

**TABLE 2:** Overview of metabolite ions which contribute the most to the predictability of the orthogonal partial least squares discriminant analysis model.

**Metabolite ID**	***m/z*[M + H]^+^**	**RT (min)**	**Predicted elemental formula**	**Matching fragments**	**Candidate metabolite with highest score**
2193	301.12560	3.69	C_1__4_H_2__0_O_7_	8/17	Rhodosin (tyrosol glycoside)
2354	317.09949	3.43	C_1__0_H_2__1_O_9_P	13/20	3-O-Butyl-alpha-D-glucopyranose 1-phosphoric acid
2778	349.14898	5.03	C_1__5_H_2__4_O_9_	17/20	Leonuridine (iridoid glycoside)
3194	388.12930	3.9	C_2__2_H_1__7_N_3_O_4_	13/18	4-[[2-(4-Methoxyphenyl)-3H-benzimidazol-5-yl]carbamoyl]benzoic acid
3435	412.06369	4.06	C_1__2_H_1__9_N_3_O_9_P_2_	16/21	N-[bis(Dimethoxyphosphoryl) methylideneamino]-2-methoxy-4-nitroaniline
3444 (NH_4_^+^)	412.21710	4.34	C_1__7_H_3__0_O_10_	19/26	((Z)-3-Hexenyl-O-α-L-arabinopyranosyl-(1,6)-β-D-glucopyranoside
3567	424.21723	5.13	C_1__8_H_3__3_NO_10_	17/21	β-Anthropyranosyl-(1→3)-α-L-rhamnopyranose
3666	445.14753	5.18	C_2__3_H_2__4_O_9_	12/20	3-(Benzofuran-2-yl)-1-[2-hydroxy-6-[(2S,3R,4S,5S,6R)-3,4,5-trihydroxy-6-(hydroxymethyl)tetrahydropyran-2-yl]oxy-phenyl]propan-1-one
3724	456.20940	4.31	C_1__9_H_2__9_N_5_O_8_	15/21	tert-Butyl N-[1-[[(2R,3S,4R,5R)-5-(4-amino-2-oxopyrimidin-1-yl)-3,4-dihydroxyoxolan-2-yl]methylamino]-1,5-dioxopentan-2-yl]carbamate
3758	461.23476	6.03	C_2__2_H_3__6_O_10_	8/20	(2E,4E)-8-(beta-D-Glucopyranosyloxy)-2,7-dimethyl-2,4-dodecadiene-1,10-dicarboxylic acid
3770	464.24035	6.06	C_2__4_H_2__9_N_7_O_3_	16/20	(2S)-2-[[(2S)-4-Cyclohexyloxy-2-[(2-methylpropan-2-yl)oxycarbonylamino]-4-oxobutanoyl]amino]-3-phenylpropanoic acid
4208	609.11274	3.99	C_2__5_H_2__5_N_2_O_1__4_P	4/18	Benzyl [[(2S,3S,4R,5R)-3-benzyloxycarbonyloxy-5-(2,4-dioxopyrimidin-1-yl)-4-hydroxy-tetrahydrofuran-2-yl]-phosphonooxy-methyl] carbonate
4302	771.16467	4.18	C_2__6_H_3__2_N_1__0_O_1__4_P_2_	3/20	[[(2R,3S,4R,5R)-5-[6-amino-8-(4-Methoxyphenyl)purin-9-yl]-3,4-dihydroxy-tetrahydrofuran-2-yl]methoxy-hydroxy-phosphoryl] [(2R,3S,4R,5R)-5-(3-carbamoylpyridin-1-ium-1-yl)-3,4-dihydroxy-tetrahydrofuran-2-yl]methyl hydrogen phosphate

**The metabolites are listed with their metabolite ID, mass over charge ratio (m/z) for the positive ionization mode, and retention time (RT). The predicted elemental formulas and in silico identifications were computed using the software package Sirius^*TM*^ and the web application MetFrag^*TM*^ Web beta. The numbers of the matching fragments from the high-resolution tandem mass spectrometry analysis are listed for the candidate metabolite with the highest score.**

Among the metabolites, several have been described to be implicated in plant defense. Metabolite 2193 (C_1__4_H_2__0_O_7_) was tentatively identified as rhodosin, a glycoside of tyrosol, a phenolic antioxidant. This compound was significantly induced in the Z-3-HAC + Fg treatment compared to the sole Z-3-HAC and Fg treatment (Figure 7). Metabolite 2778 (C_1__5_H_2__4_O_9_) was tentatively identified as leonuridine. Those metabolites for which no glucose fragment ions were generated upon HRMS/MS analysis were predicted to be phosphorylated: 3435 (C_1__2_H_1__9_N_3_O_9_P_2_), 4208 (C_2__5_H_2__5_N_2_O_1__4_P), and 4302 (C_2__6_H_3__2_N_1__0_O_1__4_P_2_). Additionally, based on the accurate mass of the [M + NH_4_]^+^ adduct of metabolite 3444 (C_1__7_H_3__0_O_10_, *m/z* value of 412.21765) and HRMS/MS spectrum (fragment ions with mass 263, 295, 233, and 245 Da as well as fragments that are linked to the sugar groups reported in the study of Sugimoto et al. (2014) (Supplementary Dataset 1), metabolite 3444 was identified as HexVic. This metabolite has been reported in the study of Sugimoto et al. (2014), in which 24 different plant species were exposed to Z-3-HOL and found to have an increased production of this diglycoside compound.

**FIGURE 8:**
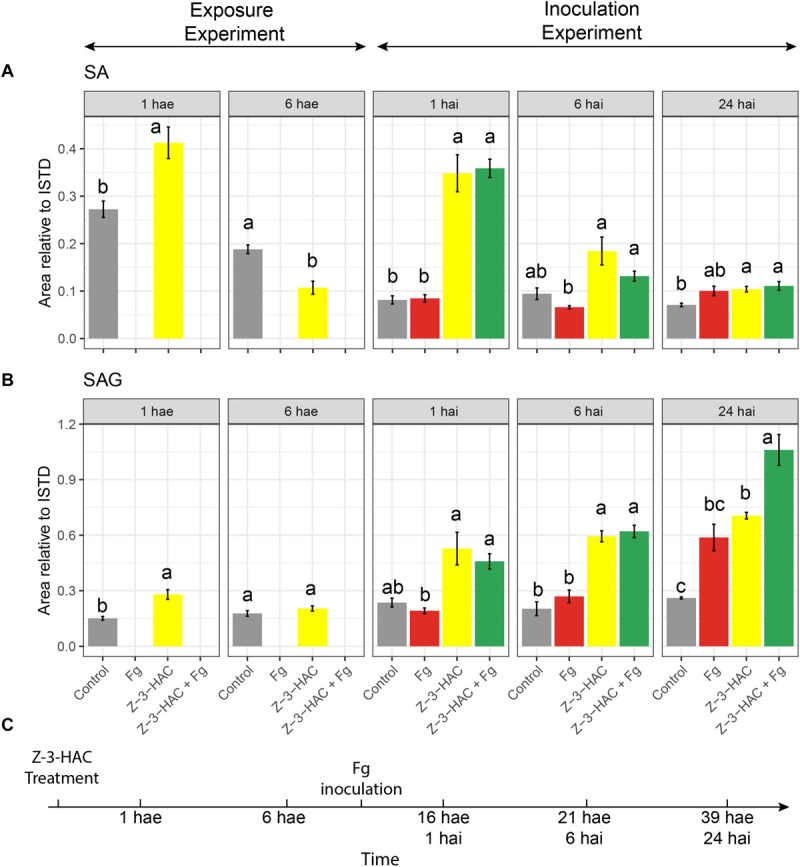
**(A)** Salicylic acid and **(B)** salicylic acid 2-O-β-D-glucoside; bars represent integrated peak area relative to the internal standard of seedlings at 1 and 6 h after exposure (hae) to Z-3-HAC and at 1, 6, and 24 h after a combination of exposure to Z-3-HAC and inoculation with a conidia suspension (10^5^ conidia mL^–1^) of *F. graminearum* (*Fg*). **(C)** Timeline of the bioassays. Bars represent the average values of 6–8 biological replicates. Treatments: control (gray bars); Fg, *F. graminearum* inoculation (red bars); Z-3-HAC, Z-3-hexenyl acetate exposure (yellow bars); Z-3-HAC + Fg inoculation (green bars); hai, hours after inoculation. Error bars represent ± SE. Significant differences between treatments per time point are depicted with different letters and calculated using a two-sample Student’s *t*-test for data at 1 and 6 hae and a *post hoc* Dunett’s T3 test for data at 1, 6, and 24 hai (α = 0.05).

We observed an increase in glycosylated metabolites upon Z-3-HAC exposure, and glycosylation of defensive compounds is one of the proposed mechanisms of priming. As glycosylation of SA is a known priming mechanism, we retrospectively analyzed the metabolome data from the exposure experiment and the metabolome data from the inoculation experiment for the presence of SA and its glycoside, salicylic acid 2-O-β-D-glucoside (SAG) (Figure 8). To this end, an authentic reference standard was purchased for identification purposes (Toronto Research Chemicals, Toronto, ON, Canada).

At 1 hae, we already saw an induction of SA biosynthesis (+ 51.4%, *P* = 0.004) in the Z-3-HAC treatment compared to control leaves. At 6 hae, the SA contents in Z-3-HAC-treated seedlings was remarkably lower compared to those of the control seedlings (−43%, *P* = 0.001). For the inoculation experiment, we observed a threefold increase in seedlings which had been treated with Z-3-HAC and Z-3-HAC + *Fg* at 1 hai (*P* < 0.01), compared to the control treatment (Figure 8). No difference was observed between the Z-3-HAC and the Z-3-HAC + *Fg* treatments. Also, at 6 and 24 hai, the SA content was higher in Z-3-HAC and Z-3-HAC + *Fg*-treated seedlings, with significant differences at 24 hai, compared to the control. At 1 hae, SAG was significantly higher (+ 85.3%, *P* = 0.003), whereas at 6 hae SAG was not significantly different compared to the control treatment (Figure 8B). At 1 and 6 hai, SAG was higher in Z-3-HAC-treated plants, but not significantly different from the control treatment. At 24 hai, SAG was significantly higher (*P* = 0.014) in the Z-3-HAC + Fg treatment compared to those of the other treatments.

## Discussion

### Z-3-HAC Induces Oxidative Stress and Transient SA Accumulation in Wheat

The notions that GLVs are quickly produced upon wounding (Fall et al., 1999) and that plants exposed to GLVs show the same activation patterns as plants which are wounded (Dombrowski and Martin, 2018; Dombrowski et al., 2019) fit within the framework of looking at GLVs as local “damaged-self signals” (Heil, 2014). Indeed GLVs are also released upon insect herbivory and fungal infection and quickly reach non-damaged parts of the plant (Ameye et al., 2018). This may then serve as a cue to induce plant defense responses against (a) biotic stress. To investigate the possible involvement of ROS in response to Z-3-HAC exposure, we performed several experiments.

We found the negative effects of 50 μM of Z-3-HAC on wheat leaves (Figures 1A,B). Remarkably, DAB staining revealed that H_2_O_2_ accumulation occurred around the stomata of the top of the leaves, which suggests that Z-3-HAC is taken up by the stomata and induces oxidative stress which is only limited to the top of the leaves. RT-qPCR analysis revealed an upregulation of genes *TaRBOH1* and *TaRBOH3* encoding for ROS-producing proteins and a suppression of genes coding for ROS-quenching enzymes. However, the enzyme activity of CAT and SOD was increased in Z-3-HAC-treated seedlings (Figure 2), which suggests that ROS quenching at these early time points is post-transcriptionally regulated and shows a delayed response. The induction of ROS and the parallel decreased expression of *CAT*, *APX*, *GPX*, and *SOD* isoforms are reminiscent of a hypersensitive response (HR) in which ROS accumulation leads to a type of programmed cell death of a number of cells to protect the plant from further biotic damage (De Gara et al., 2003; Balint-Kurti, 2019). Furthermore, this accumulation of ROS is an important hallmark to induce systemic acquired resistance (Durrant and Dong, 2004). We speculate that, during these early time points following GLV exposure, the expression of ROS-generating genes is induced, whereas the gene expression of ROS-quenching enzymes is negatively regulated, permitting the HR to carry on. However, at the protein level, the enzymes which are already present in the cells are post-transcriptionally activated (Rhee et al., 2005; Yamakura and Kawasaki, 2010), resulting in increased activity.

Pursuing a more holistic approach in which we aimed at uncovering pathways which are affected following Z-3-HAC exposure, we observed a significant effect of treating wheat with Z-3-HAC on the phenylpropanoid metabolism. This is an important pathway in plants, yielding metabolites involved in (oxidative) stress responses such as flavonoids and hydroxycinnamic acids (Dixon et al., 2002). Additionally, other metabolic pathways which also play a role in the quenching of oxidative stress, such as glutathione and ascorbate, were also affected (Figure 3). However, if Z-3-HAC-treated plants were subsequently inoculated with Fg, we observed a different effect in the phenylpropanoid pathway compared to a sole Z-3-HAC or Fg treatment (Figure 4). However, whether this effect is due to the higher oxidative stress following infection in Z-3-HAC-treated plants or related to other processes should be further investigated in future research.

Besides accumulation of ROS, our results also disclosed a link between Z-3-HAC exposure and SA production (Figure 8A). Other studies have already shown that GLVs induce an upregulation of the expression of SA and JA biosynthesis genes (Bate and Rothstein, 1998; Arimura et al., 2001; Gomi et al., 2003; Farag et al., 2005; Kishimoto et al., 2006; Engelberth et al., 2007) during the first 6 hae. The effects of Z-3-HAC on SA levels and JA-dependent defense are not mutually exclusive as GLVs have been associated with both JA and SA responses (Ameye et al., 2018). This was shown in a transcriptomics study by Mirabella et al. (2015) who demonstrated the response of *Arabidopsis* after exposure to E-2-hexenal (E-2-HAL) during the first 3 h and reported that the differentially expressed genes showed a 49% overlap with the gene expression after exposure to SA, whereas there was also a 13% overlap after JA treatment. This suggests that E-2-HAL might activate SA- and JA-dependent responses, corroborating their findings. However, this does not entail that GLV signaling is solely SA or JA dependent as Mirabella et al. (2015) found that 32% of the differentially regulated genes were unique for E-2-HAL treatment. Thus, for our model system, a holistic transcriptomics approach at these early time points will be necessary to disclose whether the early increase in SA coincides with an early increase in SA defense gene expression and whether Z-3-HAC influences defense genes downstream of SA-dependent defense signaling.

### Z-3-HAC Induces Glycosylation of Metabolites Upon Infection With *F. graminearum*

While early responses of plants upon GLV exposure have been elucidated (Asai et al., 2009; Zebelo et al., 2012), mechanisms following these responses which contribute to increased defense remain less well understood (Ameye et al., 2018). In previous research, we have already determined that Z-3-HAC induces primed defense responses in wheat (Ameye et al., 2015). However, the mechanisms through which this primed state occurred still need to be further elucidated. The accumulation of conjugated defensive compounds is one of the proposed mechanisms for defense priming. Defense-related compounds such as ABA, SA, benzoxazinoids, and phytoanticipins, among others, can be glycosylated and transported to the vacuole, rendering them inactive. Following stress triggers, these can be quickly released from the vacuole and returned to their active state through hydroxylation by glucosidases (Conrath, 2011; Pastor et al., 2014). Our data are in line with this mechanism, as we observed an increase of metabolites upon exposure to Z-3-HAC, which shared several fragment ions with D-glucose (Supplementary Dataset 1 and Supplementary Figure 3), indicating that Z-3-HAC induced the accumulation of glycosylated compounds. Furthermore, as some of these glycosylated metabolites were higher in the Z-3-HAC + Fg treatment, compared to the Z-3-HAC treatment [e.g., metabolites 2193 (C_1__4_H_2__0_O_7_), 2354 (C_1__0_H_2__1_O_9_P), and 3567 (C_1__8_H_3__3_NO_10_)], these metabolites may point to mechanisms involved in the primed defense response of wheat plants following Z-3-HAC exposure.

Glycosyltransferases (GTs) play an important role in the biosynthesis and maintenance of the cell wall (Scheible and Pauly, 2004). GTs are also involved in plant defense by detoxifying xenobiotic compounds, such as mycotoxins (Lulin et al., 2010; Audenaert et al., 2013). Furthermore, GTs play a role in stabilizing and increasing the solubility of plant-defensive compounds such as phyto-anticipins, plant defense hormones, and their precursors. After glycosylation, these compounds can be transported and stored in the vacuole, from which they can be released upon stress or cellular damage and transformed into their active aglycons by glucosidases, which are located in different cell organelles (Bowles et al., 2006; Morant et al., 2008; Pastor et al., 2013). GTs constitute a large group of enzymes, with a restricted substrate specificity, entailing that different GTs glycosylate specific compounds (Jones and Vogt, 2001; Hansen et al., 2012). Several metabolites shared the same HRMS/MS fragment ions (Supplementary Figure 3), suggesting that some metabolites may have identical or similar aglycone substructure and thus may originate from the same, yet unknown, pathway(s). For example, metabolite 2193 has been identified as leonuridine, a glycosylated terpenoid, a member of the iridoid glycosides which exhibits antioxidant properties (Dinda et al., 2011). Leonuridine has additionally been reported to increase in *Quercus suber* following drought stress (Almeida et al., 2020). However, metabolite 2354 (C_1__0_H_2__1_O_9_P) shares several fragment ions (Supplementary Dataset 1) and exhibits an identical pattern over time (Figure 7), which suggests that these may originate from the same pathway. Another glycoside was identified in our metabolomics study to be significantly produced upon Z-3-HAC exposure, the hexenyl diglycoside HexVic. The presence of hexenyl glycosides in plants has already been described in literature, whereby these compounds have primarily been reported to act as precursors for Z-3-HOL production (Jae-Hak et al., 1996; Nishikitani et al., 1999; Sugimoto et al., 2015). Moreover, it has been shown that one of the early responses (60 min) of maize following Z-3-HOL exposure involves the upregulation of putative glucosyltransferases and UDP-glucoside hydrogenase, which may result in concentration changes of glycosylated compounds (Engelberth et al., 2013). In a later study by Sugimoto et al. (2014), it has been shown for 24 different plant species, including *Arabidopsis* and *Triticum aestivum*, that hexenyl glucosides and hexenyl diglucosides increased after exposure to Z-3-HOL. The enzyme responsible for the synthesis of Z-3-hexenyl glycoside in *Camellia sinensis* was identified. Ohgami et al. (2015) demonstrated *in vitro* that AtUGT85A3 produced hexenyl-glycosides with UDP-glucose as a sugar donor and Z-3-hexenol as a sugar acceptor. Furthermore, they showed that homologs of the UGT85 family in several other plant species also formed hexenyl-glycosides. CsGT1, the AtUGT85A3 homolog in *C. sinensis*, was shown to have broad substrate specificity, including benzyl alcohol, linalool, geraniol, and Z-3-hexenol. However, GTs preferentially bind to hydroxyl groups, implying that, in our study, Z-3-HAC most likely must be hydrolyzed to Z-3-HOL by an esterase before it could be glycosylated.

We specifically investigated whether glycosylation of SA was also induced in Z-3-HAC-treated wheat. Indeed Z-3-HAC triggered an increased production of SAG (Figure 8B), corroborating that the defense-inducing action of GLVs may be attributed to the accumulation of glycosylated defensive compounds. These glycosylated compounds may be stored in the vacuole or transported through the xylem to other plant parts (Chen et al., 1995; Ratzinger et al., 2009). The time course of SA and its glycosylated derivate SAG accumulation follows the pattern for a priming response as stated by Martinez-Medina et al. (2016), i.e., following a priming stimulus (Z-3-HAC exposure), there is a transient induction of SA and SAG accumulation at 1 hae which returns to control levels at 6 hae, in the absence of a stress trigger (Figures 8A,C). However, following leaf sheath inoculation, a stress stimulus arises (wounding response because of the peeling of the leaf sheath and/or Fg inoculation), during which we observed a higher induction of SA and SAG formation in the Z-3-HAC and Z-3-HAC + Fg treatment at 1 and 6 hai. At 24 hai, SAG in the Z-3-HAC + Fg treatment is significantly higher than in the Z-3-HAC and the Fg treatment (Figure 8B), underlying the hypothesis that exposure to Z-3-HAC primes plant defense by glycosylating plant defense compounds.

These data together suggest that GLVs induce a transient production of defense signaling compounds (SA and SAG) on the first hours after exposure. After an (a)biotic stress trigger in that tissue, SA and SAG formation are more strongly induced compared to plant tissue which was not previously exposed to Z-3-HAC. Then, Z-3-HAC directly induces glycosylation processes, which may play a role in plant defense. These glycosylation processes are activated upon Z-3-HAC perception and are even more enhanced when Z-3-HAC-treated plants encounter an (a)biotic stress trigger.

## Conclusion

In conclusion, using a combined approach of the RT-qPCR analysis together with the metabolome data may point to a role for Z-3-HAC in the defense against pathogens by eliciting a ROS burst and increased activity of ROS-scavenging enzymes. This resulted in the activation of several pathways which are related to antioxidative processes. Indeed ROS production leading to HR constitutes a crude but effective means to fend off aggressors during the biotrophic phase of its infection phase in which living cells are needed to successfully infect the plant tissue. Additionally, our metabolomics approach showed an increase in glycosylation processes following Z-3-HAC exposure. However, further research is mandatory to elucidate the full identity of these glycosylated compounds which are produced more upon a combined treatment in order to unravel whether these involved plant defense.

## Data Availability Statement

The raw data supporting the conclusions of this article will be made available by the authors, without undue reservation, to any qualified researcher.

## Author Contributions

MA, GS, GH, KA, LM, and LV designed the experiments. MA performed all the experiments and wrote the manuscript. BM helped with the ROS experiment. LM and LV provided the valuable input for the metabolomics experiments. All authors read and revised the manuscript.

## Conflict of Interest

The authors declare that the research was conducted in the absence of any commercial or financial relationships that could be construed as a potential conflict of interest.

## Supplementary Material

Supplementary Figure 1 Levels of L-Phe in the infection assay.

Supplementary Figure 2 Predicted chemical structures of the metabolites which contribute the most to the predictability of the orthogonal partial least squares discriminant analysis model.

Supplementary Figure 3 High-resolution tandem mass spectrometry patterns of the metabolites which contributed most to the predictability of the orthogonal partial least squares discriminant analysis model for the positive ionization mode.

Supplementary Figure 4 Principal component analysis score plots for the different time points of the negative ionization mode.

Supplementary Figure 5 S-plot of the metabolite features for the samples at 6 h after inoculation, making the comparison between the Z-3-HAC and Z-3-HAC + Fg treatments.

Supplementary Table 1 Accurate mass, ionization adduct, and retention time as determined based on analytical standards and used for the identification of salicylic acid.

Supplementary Dataset 1 Excel datasheet containing fragment ions of metabolites which were most abundant in the Z-3-HAC and Z-3-HAC + Fg treatments.
