# Supplementary material for: Metabolomics Reveal Induction of ROS Production and Glycosylation Events in Wheat Upon Exposure to the Green Leaf Volatile Z-3-Hexenyl Acetate
Source: Front Plant Sci. 2020 Dec 3;11:596271. doi: 10.3389/fpls.2020.596271 (PMC7744478; doi:10.3389/fpls.2020.596271)
Supplement: Supplementary Figure 1 — Levels of L-Phe in the infection assay. [file Table_1.DOCX]

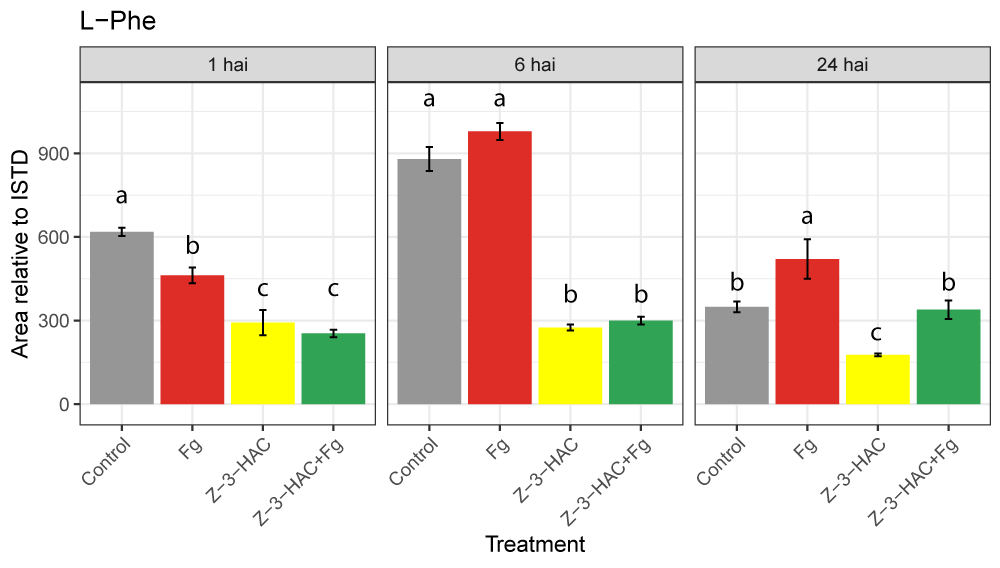


Fig**. S1** Z-3-HAC exposure leads to lower levels of L-phenylalanine, but has no effect on p-coumaric acid. Peak area relative to the internal standard (ISTD) is shown. As internal standard, a deuterium labelled analytical standard of 100 pg μl-1 d6-abscicic acid (OlChemIm, Olomouc, Czech Republic) was used. The number of biological replicates per treatment are shown beneath each bar. Each biological replicate consists of 100 mg fresh weight of pooled leaf sheats. Error bars represent SE. Significance of differences was calculated using one-way ANOVA with a post-hoc bonferonni test.


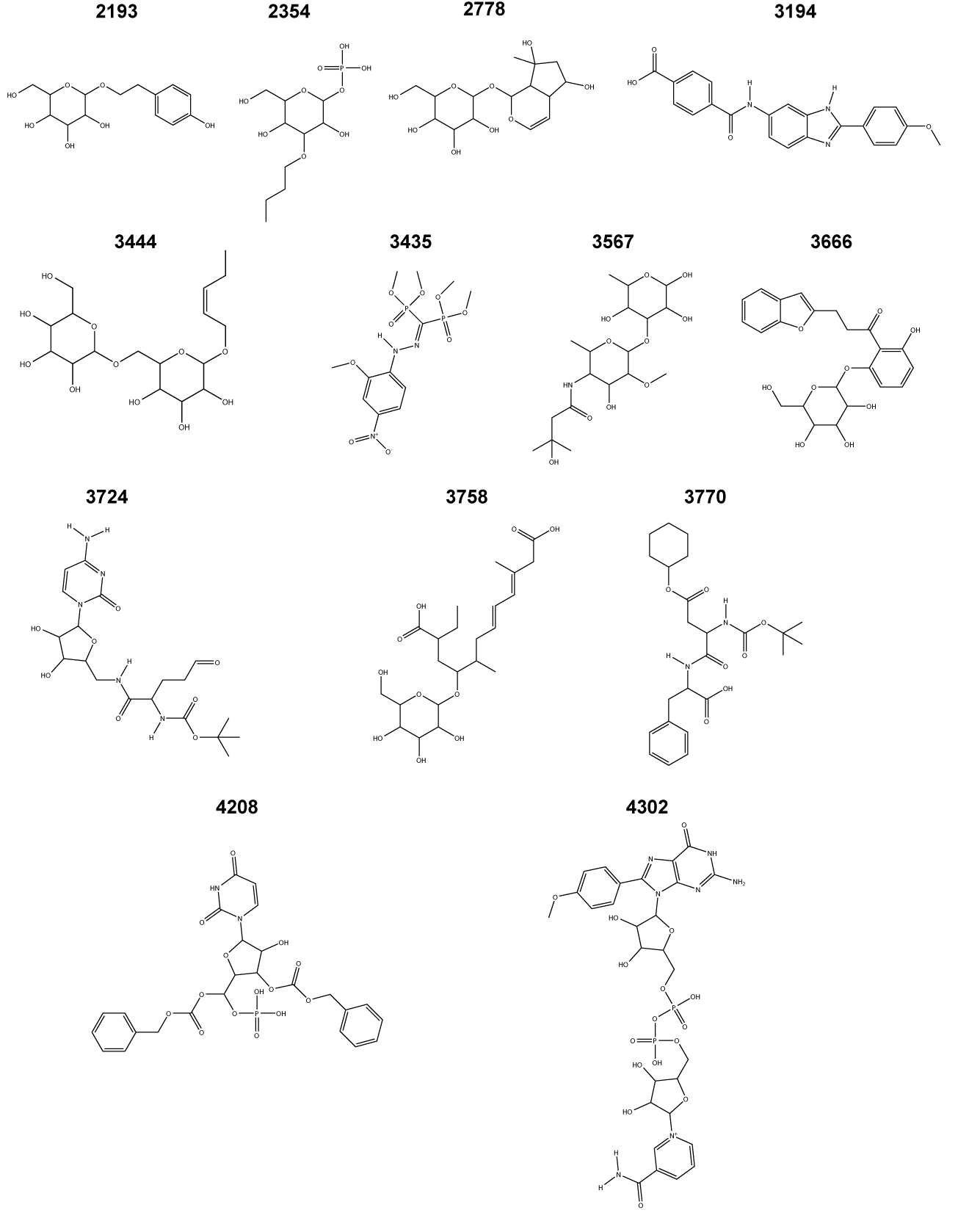


**Fig. S2** Predicted chemical structure of the metabolites which contributed the most to the predictability of the OPLS-DA model. Metabolite codes were automatically assigned by Sieve™ 2.1 software (Thermo Fisher Scientific, San Jose, USA). Chemical structures were predicted based on the accurate mass and HRMS/MS patterns, using the MetFrag web application and Sirius software. lengths (a). Broader violin plots represent values which are more frequent around those values. Error bars represent +/- SE. Plots depicted with different letters indicate significant differences. Significance of differences (α=0.05) was calculated with a two-sample t-test.


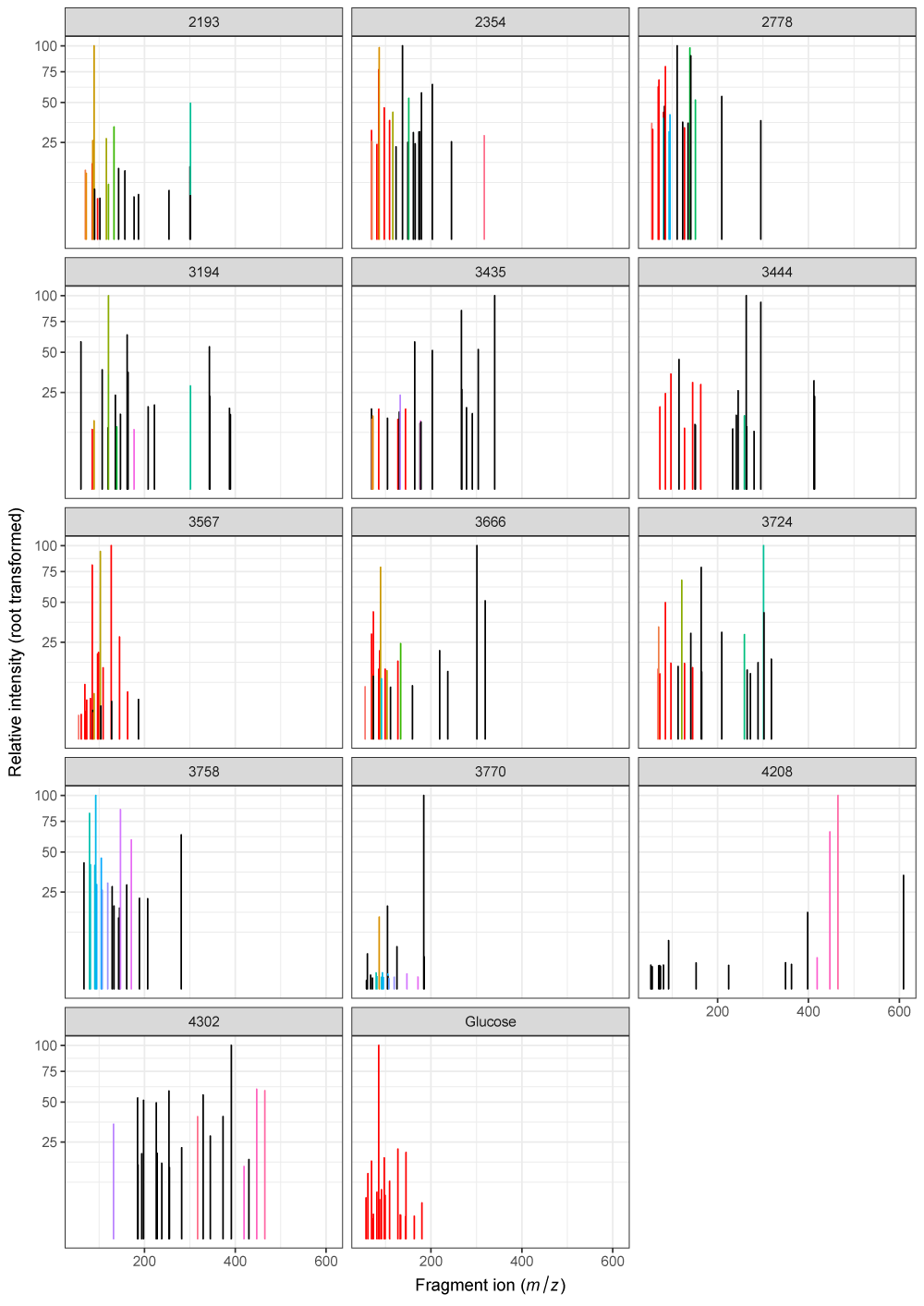


Fig. S3 HRMS/MS patterns of the metabolites which contributed most to the predictability of the OPLS-DA model for the positive ionization mode. Fragment ions matchion the the fragmentation spectrum of glucose are depicted in red. Fragments ions which are shared between metabolites are depicted in the same colour, whereas fragments which are not shared are depicted in black. A root transformation on the relative intensity was carried out to better visualize fragments which were low in relative intensity.


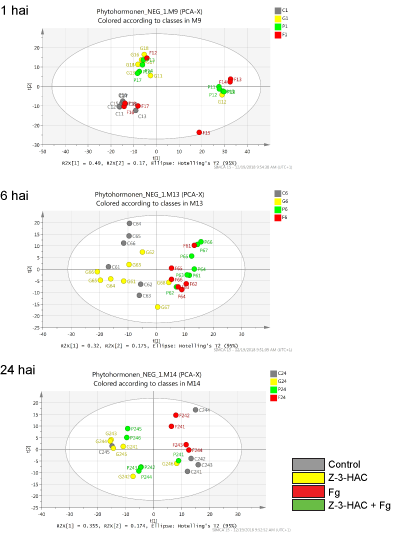


**Fig. S4** Principal Component Analysis score plots for the different time points. PCA plots were constructed with metabolome data from the positive ionization mode for different time points: 1, 6, 24 hour after inoculation (hai). Each circle represent a single sample of 100 mg of 6 to 8 pooled leaf sheaths. Control: grey, Z-3-HAC: yellow, Z-3-HAC+Fg: green, Fg: red. The ellipse depicts the Hotteling’s T^2^ 95% confidence interval.

Table S1

| **Gene** | **Forward primer (5’-3’)** | **Reverse primer (5’-3’)** | **Referentie** |
| --- | --- | --- | --- |
| ***Actin*** | CGAAACCTTCAGTTGCCCAGCAAT | ACCATCACCAGAGTCGAGCACAAT | (Wang et al., 2016) |
| ***Ta54227*** | CAAATACGCCATCAGGGAGAACATC | CGCTGCCGAAACCACGAGAC | (Paolacci et al., 2009) |
| ***CAT*** | CACCTGGTGGAGAAGATCGC | TS^1^ACCTCGAAGAAGCCCTTG | (Dudziak et al., 2019) |
| ***GPX*** | GCGGTGACACCAACATCAAC | GTCCAGGTTCK^2^CCAGGTTGG | (Dudziak et al., 2019) |
| ***APX*** | CAAGGCTCTGACCACCTCAG | CATCTTCCCAGGGTGTGACC | (Dudziak et al., 2019) |
| ***CuSOD*** | AGAAGCACCACGCCACCTA | CACCCATCCAGATCCTTGTAAAG |  |
| ***MnSOD*** | ATGGCAGGGAAACCCG | TTAAACAGCAGGTTGAATTCC | (Wang et al., 2016) |
| ***TaRboh1*** | GAGGAAGACGAGCACCAAA | TTTCCTCGCGAAAAGAGAAA | (Wang et al., 2016) |
| ***TaRboh2*** | GTCGGCAGATTTCACCCA | CCTGATGAAGCATGGCATAG | (Wang et al., 2016) |
| ***TaRboh3*** | CTAACAAAGGAGCTGCGTGA | AGAAGTTAAAAATTCTCCTTGTGG | (Wang et al., 2016) |

**Dudziak, K., Zapalska, M., Börner, A., Szczerba, H., Kowalczyk, K., and Nowak, M. (2019). Analysis of wheat gene expression related to the oxidative stress response and signal transduction under short-term osmotic stress. Scientific reports 9, 1-14.**

**Paolacci, A.R., Tanzarella, O.A., Porceddu, E., and Ciaffi, M. (2009). Identification and validation of reference genes for quantitative RT-PCR normalization in wheat. BMC Molecular Biology 10.**

**Wang, M., Zhao, X., Xiao, Z., Yin, X., Xing, T., and Xia, G. (2016). A wheat superoxide dismutase gene TaSOD2 enhances salt resistance through modulating redox homeostasis by promoting NADPH oxidase activity. Plant molecular biology 91, 115-130.**

**Table S2** Accurate mass, ionization adduct and retention time as determined based on analytical standards and used for the identification of selected phytohormones.

| **Metabolite** | **Ionization adduct** | **Accurate mass *(m/z)*** | **Retention time (min)** |
| --- | --- | --- | --- |
| Salicylic acid (SA) | - H^+^ | 137.02357 | 5.05 |
| D_6_-ABA (Int. Std.) | - H^+^ | 269.16650 | 5.25 |

**
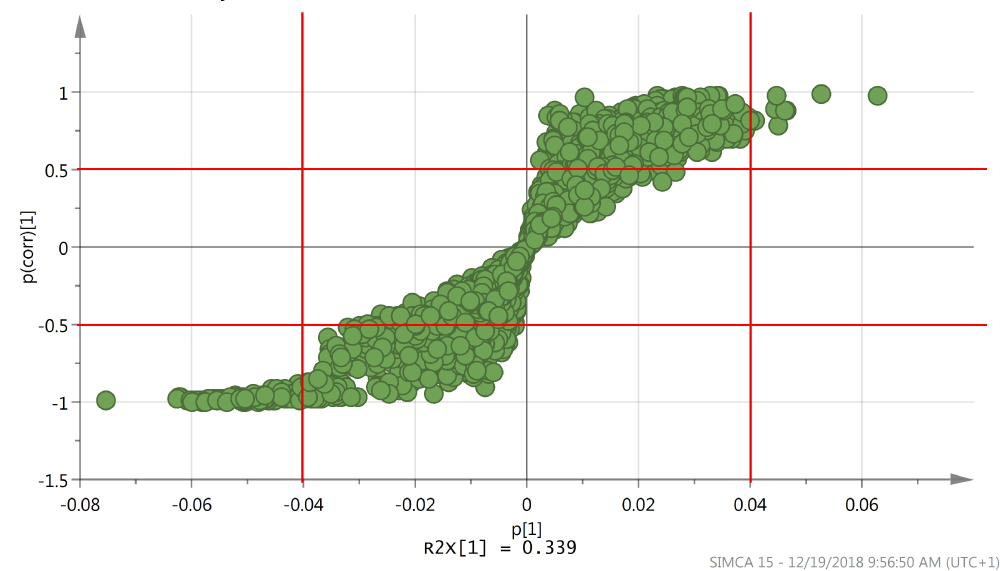
**

**Fig. S3** : S-plot of the metabolite features for the samples at 6 hours after inoculation, making the comparison between the Z-3-HAC and Z-3-HAC+Fg treatment. Red lines indicate the covariance and |correlation p(corr)| cut-off values to retain metabolite features for further analysis.
